# Anticancer effect of icaritin on prostate cancer via regulating miR‐381‐3p and its target gene UBE2C

**DOI:** 10.1002/cam4.2630

**Published:** 2019-10-24

**Authors:** Jimeng Hu, Xiaobo Wu, Chen Yang, Khalid Rashid, Chenkai Ma, Mengbo Hu, Qiang Ding, Haowen Jiang

**Affiliations:** ^1^ Department of Urology Huashan Hospital Fudan University Shanghai China; ^2^ Department of Medical Oncology Zhongshan Hospital Fudan University Shanghai China; ^3^ Department of Surgery Royal Melbourne Hospital University of Melbourne Melbourne Vic. Australia

**Keywords:** icaritin, prostate cancer, TRAMP, UBE2C

## Abstract

Prostate cancer (PCa) is one of the most common health‐related issues in the male individuals of western countries. Icaritin (ICT) is a traditional Chinese herbal medicine that exhibits antitumor efficacy in variety of cancers including PCa. However, the precise function and detailed molecular mechanism of ICT in the regression of PCa remain unclear. Ubiquitin‐conjugating enzyme E2C (UBE2C) is an anaphase‐promoting complex/cyclosome (APC/C)‐specific ubiquitin conjugating enzyme, which acts as an oncogene in PCa progression. The function of ICT in PCa was investigated in transgenic adenocarcinoma mouse prostate (TRAMP) mice using survival analysis, hematoxylin and eosin (HE) staining, TUNEL assay, and immunohistochemistry and in human PCa cell lines using various molecular techniques and functional assays including plasmid construction and transfection. Bioinformatic analyses were performed to identify the interaction between miRNA and UBE2C via the TargetScan algorithm. We demonstrated that ICT inhibited the development and progression of PCa in TRAMP mice by improving the survival rate and tumor differentiation. Furthermore, we found that ICT could significantly inhibit cell proliferation and invasion and induce apoptosis in PCa cells. Consistently, downregulation of UBE2C suppressed the proliferation and invasion of PCa cells. Moreover, a luciferase reporter assay confirmed that UBE2C was a direct target of miR‐381‐3p. Meanwhile, ICT simultaneously downregulated UBE2C expression and upregulated miR‐381‐3p levels in human PCa cells. Altogether, our findings provide a strong rationale for the clinical application of ICT as a potential oncotherapeutic agent against PCa via a novel molecular mechanism of regulating the miR‐381‐3p/UBE2C pathway.

## Graphical abstract

We demonstrated that ICT inhibited the development and progression of PCa in TRAMP mice, by improving survival and tumor differentiation. Our results provide a strong rationale for the potential clinical application of ICT as a novel therapeutic agent against PCa via a potential mechanism of regulating the miR‐381‐3p/UBE2C pathway.

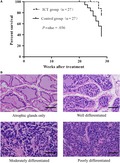

## 1 INTRODUCTION

Prostate cancer (PCa) is one of the most fatal malignancies being common among men in western countries.^1^ The incidence rates of PCa have also dramatically increased in Asian populations, especially in developed metropolitan areas.^2^ For patients with locally advanced PCa, androgen deprivation therapy (ADT) is the standard treatment. Although the initial response rate of ADT is high (70%‐80%), the therapeutic resistance, castration resistance, is evitable in most of PCa.^3^ Hence, there is a need to a novel alternative treatment to ADT after resistance is induced.

Ubiquitination participants in numerous cellular biological processes, including the degradation of short‐lived proteins in various cell types. Ubiquitin‐conjugating enzyme E2C (UBE2C), working closely with anaphase‐promoting complex/cyclosome (APC/C), is one of the important enzymes for ubiquitination.^4^ The aberrantly high expression of UBE2C may result in tumorigenesis and potentially be a prognostic biomarker for cancer.^5^ The elevated expression of UBE2C was detected in various human solid cancers, such as colon,^6^ lung,^7^ liver,^8^ breast,^9^ and thyroid.^10^ Furthermore, overexpression of UBE2C was also found in PCa,^11^, ^12^ especially in castration‐resistant PCa (CRPC).^13^, ^14^ However, the role of UBE2C in CRPC still remains unclear.

On the one hand, the expression of UBE2C could be regulated by microRNAs (miRNAs) in various types of cancer.^15^, ^16^ Abnormal expression of miRNAs has been found to be associated with various diseases including tumorigenesis. MiRNAs can act as either tumor suppressor genes or oncogenes depending on the target mRNAs in various tumor types, including PCa.^17^ For instance, miR‐16 inhibits the growth of metastatic PCa via downregulating multiple cell cycle genes whereas miR‐141 and miR‐375 enhance the PCa progression.^18^, ^19^ Furthermore, Zhang et al^20^ demonstrated that miR‐381 functioned as a tumor suppressor microRNA in rectal carcinoma through specific inhibition of UBE2C expression.

Icaritin (ICT) is a Chinese herbal medicine which shows a range of different biological and pharmacological functions including the induction of cardiac differentiation in mouse embryonic stem cells,^17^ stimulation of neuronal differentiation,^18^ and inhibition of hematological malignancy growths.^19^, ^21^ At submicromolar levels, ICT shows increased estrogen‐like activity in estrogen receptor‐positive breast cancer MCF‐7 cells,^22^ whereas it inhibits the growth of renal cell carcinoma at micromolar concentrations.^23^ In line with these findings, we have previously illustrated that ICT not only increases the survival rate of transgenic adenocarcinoma mouse prostate (TRAMP) mice but also inhibits the growth of prostate cancer cell line LNCaP.^24^, ^25^ However, the specific targets and the underlying mechanisms of ICT in suppressing PCa progression have not yet been well‐investigated.

Herein, we hypothesized that ICT has an antitumor effect on PCa via miRNA‐381‐3p and its target UBE2C. In vivo and in vitro effects of ICT treatment were investigated in TRAMP mice and PCa cell lines, respectively. ICT was found to exert growth inhibitory effects on PCa cells. We also demonstrated that ICT inhibits the malignant transformation of PCa by regulating miR‐381‐3p targeting UBE2C gene. These results indicate a novel application of ICT as a potential antitumor agent in PCa treatment.

## 2 MATERIALS AND METHODS

### 2.1 Animal models and tissue specimens

This study was performed in accordance with the proposal of “Institutional Animal Care and Use Committee from Huashan Hospital at Fudan University.” The protocol was approved by this committee. TRAMP mice were received from Jackson Laboratory. Generation of transgenic mice, isolation of mouse‐tiptoe DNA, and PCR‐based screening assays were carried out as previously described.^26^ A total of 54 TRAMP mice were randomly divided into two groups (experimental group and control group). In the experimental group, TRAMP mice received an intraperitoneal injection of ICT solution at a dose of 30 mg/kg five times per week, while the control group received the same volume of saline solution. Each group received the treatments from 8 weeks of age until scheduled killing.

TRAMP mice from both groups were separated into three subgroups, which were plan to be killed on the 20th, 24th, and 28th week, through asphyxiation with CO_2_. Each subgroup contained nine mice. TRAMP mice were required to fast overnight before being sampled. Both prostate tissue and serum were kept frozen at −80°C.

### 2.2 Histopathological studies

Paraffin‐embedded prostate tissues were cut into 10 μm sections and prepared for hematoxylin and eosin (HE), immunohistochemical, and Terminal deoxynucleotidyl transferase‐mediated dUTP nick end labeling (TUNEL) staining. Histopathology evaluation was accomplished by two independent observers to distinguish tumor tissue differentiation grade, as we described previously.^23^ Consecutive sections from one of the six replicate blocks were used for immunohistochemical staining. Percentage of Ki‐67‐positive cells and densities of UBE2C and TUNEL staining were quantified using Image‐Pro Plus software, each with five randomly selected fields. The primary antibodies against UBE2C or Ki‐67 (Santa Cruz Biotechnology) at the recommended dilutions were dropped into each slide and incubated overnight at 4°C. Normal IgG was applied as a negative control. Then, each slide was washed with PBS and incubated at room temperature for 2 hours. The TUNEL assay was carried out using the TUNEL BrightGreen Apoptosis Detection Kit (Vazyme Biotech Co.), following the manufacturer's instruction.

### 2.3 Cell culture and ICT treatment

Human PCa cell lines LNCaP and PC‐3 were purchased from the American Type Culture Collection (ATCC). Cells were cultured in RPMI‐1640 medium (Gibco) combined with 10% fetal bovine serum (FBS; Gibco) and penicillin‐streptomycin (Gibco) at 37°C in a humidified atmosphere with 5% CO_2_. Cells were treated with indicated concentrations of ICT with a purity of up to 99.5% (Shanghai Win Herb Medical Science Corporation) for the indicated time points. ICT was dissolved in DMSO (sigma), and the final concentration of DMSO in the working solution of ICT was restricted to less than 0.1% of total medium volume.

### 2.4 Transfection of miRNA

The miR‐381‐3p mimic and another scramble oligonucleotide, named miR‐381‐3p and miR‐NC, respectively, were synthesized by RiboBio biotechnology. MiRNA transfection was performed with lipofectamine 2000 reagent (Invitrogen), according to the manufacturer's instruction.

### 2.5 Plasmid construction and shRNA transfection

The plasmid and short hairpin RNAs (shRNAs) used for transfection of UBE2C were obtained from GeneChem Co. Ltd. For viral infection, cells with 70%‐80% confluence were infected with the lentivirus vector and 4 mg/mL polybrene (Sigma‐Aldrich). For cell transfection, cells with 80% confluence were transfected through Lipofectamine 2000 reagent in Opti‐MEM medium (Invitrogen). At 48 hours post‐transfection, cells were harvested for further studies.

### 2.6 Cell proliferation assay

Cell viability was assessed using the Cell Counting Kit‐8 (CCK‐8; Dojindo) assay. PCa cells (2 × 10^4^ per well) were plated in 96‐well plates and incubated with different concentrations of ICT solution or transfected with UBE2C shRNA for overnight.

### 2.7 Cell apoptosis assay

The apoptotic cell death was evaluated by annexin‐V FITC/PI double staining. Cells were harvested and washed with PBS twice, resuspended with 100 μL of binding buffer, and stained with 5 μL of annexin‐V FITC and 10 μL of PI in the dark at room temperature for 15 minutes. Then, each solution was diluted with 300 μL of binding buffer. The percentage of apoptotic cells was analyzed by FACScan flow cytometry (Becton Dickinson) and the data were analyzed using FlowJo software (Tree Star Inc).

### 2.8 Cell cycle analysis

PCa cells were treated in 6‐well plates, harvested, washed twice with ice‐cold PBS, centrifuged, resuspended, and fixed with 70% of ethanol for overnight at 4°C. Next, cells were washed and resuspended with PBS, pretreated with 10 μg/mL of RNase for 10 minutes, and stained with 100 μL of 100 μg/mL PI for 20 minutes in the dark at room temperature. Cell cycle profiles were assayed using FAC Scan flow cytometry (Becton Dickinson).

### 2.9 Cell invasion assay

Transwell chambers (Corning Inc) were used to detect cell invasion with coated Matrigel in the upper chamber. PCa cells (5 × 10^4^) were starved for 24 hours before the experiment and placed in the upper chamber with 150 μL of serum‐free medium, whereas the lower chamber was loaded with a total of 500 μL of medium containing 20% FBS. The inserts were placed in 4% paraformaldehyde for 15 minutes, stained with a 0.1% crystal violet staining solution for 30 minutes, counted in three random fields under microscopy, and photographed. OD values (absorbance at 570 nm) of the solution were detected using an enzyme‐linked immunosorbent assay reader (Bio‐Rad).

### 2.10 RNA extraction and real‐time polymerase chain reaction

Total RNA was extracted from PCa cells using Trizol reagent (Invitrogen) and the RNA was eluted in 50 μL of RNAse‐free water. The concentration of the total RNA was measured by the Nanodrop® ND‐1000 UV spectrophotometer at a wavelength of 260 nm. The RNA purity was determined by spectrophotometry using ratio of wavelength at 260 and 280 nm.

Quantitative real‐time PCR gene expression profiling was performed using cyberGreen master mix dye and detected by the ABI Prism 7300 real‐time PCR system (Applied Biosystems). The initial conditions for thermal cycling consisted of preheating at 95°C for 5 minutes followed by 40 cycles of 15 seconds at 95°C, 20 seconds at 60°C, and 20 seconds at 72°C. The relative gene mRNA expression was normalized to that of β‐actin as a loading control. The mRNA expression of UBE2C (sense:5′‐TGATGTCTGGCGATAAAGGG‐3′; anti‐sense: 5′‐TGATAGCAGGGCGTGAGGAA‐3′) and β‐actin (sense: 5′‐GGCACTCTTCCAGCCTTCC‐3′; anti‐sense: 5′‐GAGCCGCCGATCCACAC‐3′) were assessed. In addition, real‐time PCR for miR‐381‐3p was performed using the All‐in‐OneTM miRNA qRT‐PCR Detection Kit (GeneCopoeia). The expression level of miR‐381‐3p was normalized to U6 snRNA as endogenous control. Fold expression levels were calculated using the $2^{-\Delta \Delta C_{t}}$ method.

### 2.11 Luciferase reporter assay

The human UBE2C 3′UTR containing the putative miR‐381‐3p binding sites was amplified through PCR with the forward primer, 5′‐TCAGCTCGAGTGTGTCGTCTTTTTAATTTTTCCT‐3′ and the reverse primer, 5′‐AATTGCGGCCGCTTATTTAATATACAAGGGCTCAACC‐3′. The mutant UBE2C 3′UTR (5′‐AGCCUCGGUUGAGCCCUUGUUAU‐3′) with point substitutions in the miR‐381‐3p binding sites was synthesized by Invitrogen. The product was cloned into the Not I and Xho I sites of the psi‐CHECK2 luciferase reporter vector (Promega). The above constructs were sequence verified.

Human PCa cells were plated in 96‐well plates and transfected with wild‐type or mutant UBE2C 3′UTR vectors (100 ng per well), together with miR‐381‐3p or miR‐NC (30 pmol per well). After 48 hours of transfection, cells were harvested for the luciferase assay. The activities of Renilla and firefly luciferases were evaluated via Dual‐Luciferase Reporter Assay System (Promega). Values were normalized to firefly luciferase activity.

### 2.12 Protein extraction and western blot assay

After ICT treatment or transfection, cells were washed twice in cold PBS and then lysed on ice with RIPA buffer containing protease and phosphatase inhibitors (Thermo Fisher Scientific). Then, the given cell lysates were subject to run gel for separation and transferred to the members using our previously established protocols. The primary antibodies against UBE2C (sc‐166339), cyclin D1 (sc‐753) and cyclin E (sc‐247) were purchased from Santa Cruz Biotechnology. Antibody for cleaved caspase 3 (#9654) was obtained from Cell Signaling Technology, while the antibody against GAPDH was used as a loading control.

### 2.13 Statistical analysis

All statistical analyses were performed using SPSS 17.0 for Windows (SPSS Inc). The level of significance for the differences between these two groups was evaluated using Student's *t* test. The result was considered statistically significant when the *P* value was less than .05.

## 3 RESULTS

### 3.1 Effect of ICT treatment on survival and tumor differentiation in TRAMP mice

**Figure 1:**
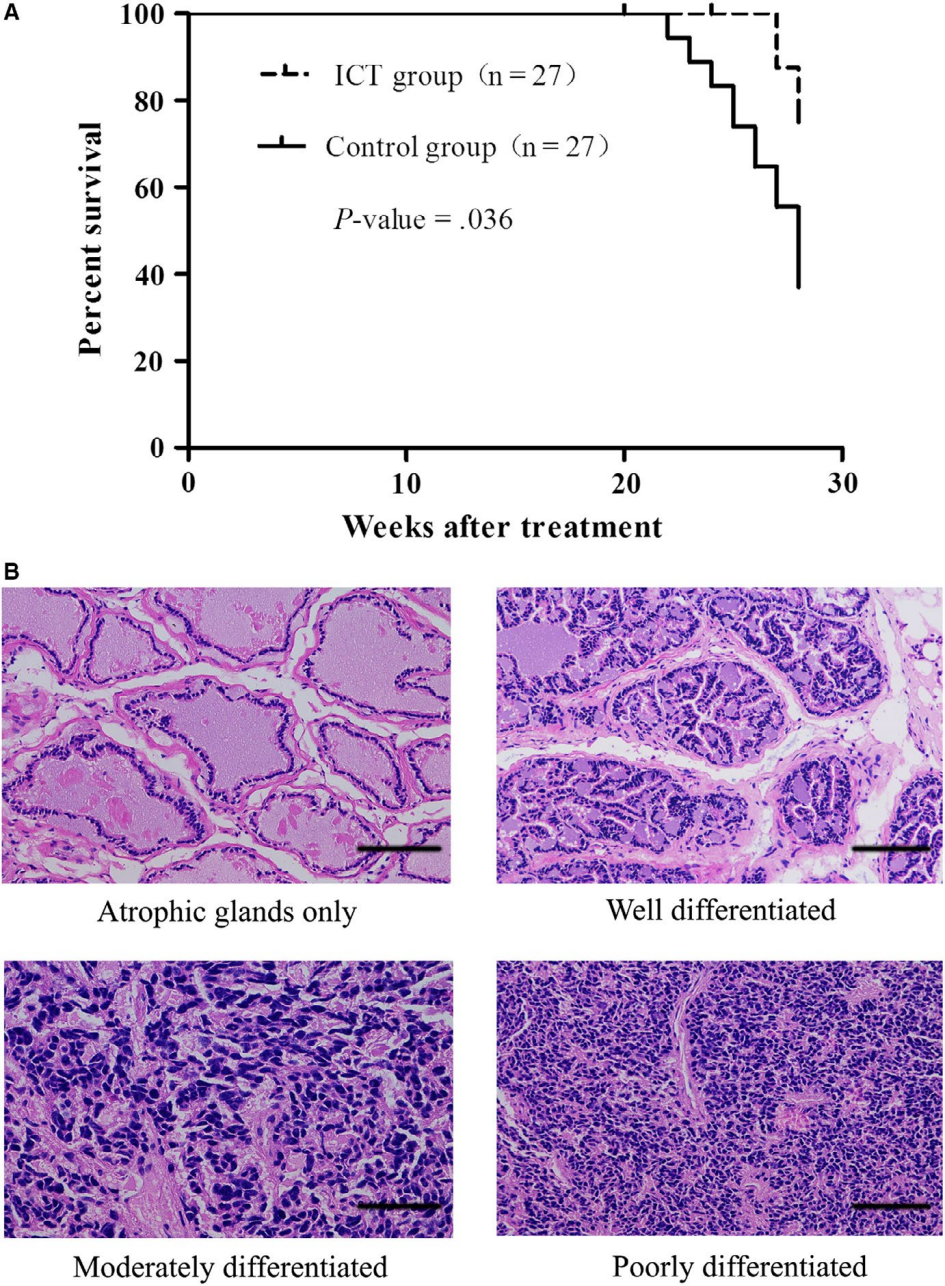
Effect of icaritin (ICT) treatment on survival and tumor differentiation in TRAMP mice. A, A significant survival increase (*P* = .036, log‐rank test) was observed in TRAMP mice from ICT treatment group. B, Representative images of different histology grades from prostatic tissue sections with HE staining (magnification, 200×; scale bar, 100 μm)

To investigate the in vivo antitumor efficacy of ICT, we delivered ICT on TRAMP mice. Our results showed that ICT treatment significantly increased the survival of TRAMP mice as compared to the survival of mice in the control group (*P* = .036, log‐rank test) (Figure 1A). However, we did not observe any significant differences of survival rates between these two groups in 28 weeks after the start of the treatments. A total of 10 TRAMP mice died from advanced PCa, where two were from the ICT group and eight were from the control group. The biological samples were collected from all TRAMP mice, including those scheduled for killing and the 10 that died prematurely.

The evaluation of PCa samples from TRAMP mice is presented in Table 1. No significant difference was observed in different histological grades of PCa (*P* > .05). The incidence of well‐differentiated tumor tissue in the ICT group (44.00%) was moderately higher than that in the control group (26.32%). Meanwhile, the incidence of poorly differentiated tumor tissue in the ICT group (20.00%) was moderately lower compared with that in the control group (42.10%), indicating that the ICT improves TRAMP mice survival by the induction of prostate cancer differentiation. Representative HE‐stained images of different histological grades are showed in Figure 1B.

**Table 1:** Prostate histopathology of TRAMP mice

Histopathology evaluation	ICT group (n = 25)	Control group (n = 19)	*P*
Atrophic glands only n (%)	3 (12.00)	1 (5.26)	.622
Well differentiated n (%)	11 (44.00)	5 (26.32)	0.344
Moderately differentiated n (%)	6 (24.00)	5 (26.32)	1.000
Poorly differentiated n (%)	5 (20.00)	8 (42.10)	.182

### 3.2 UBE2C expression were downregulated in ICT‐treated TRAMP mice

**Figure 2:**
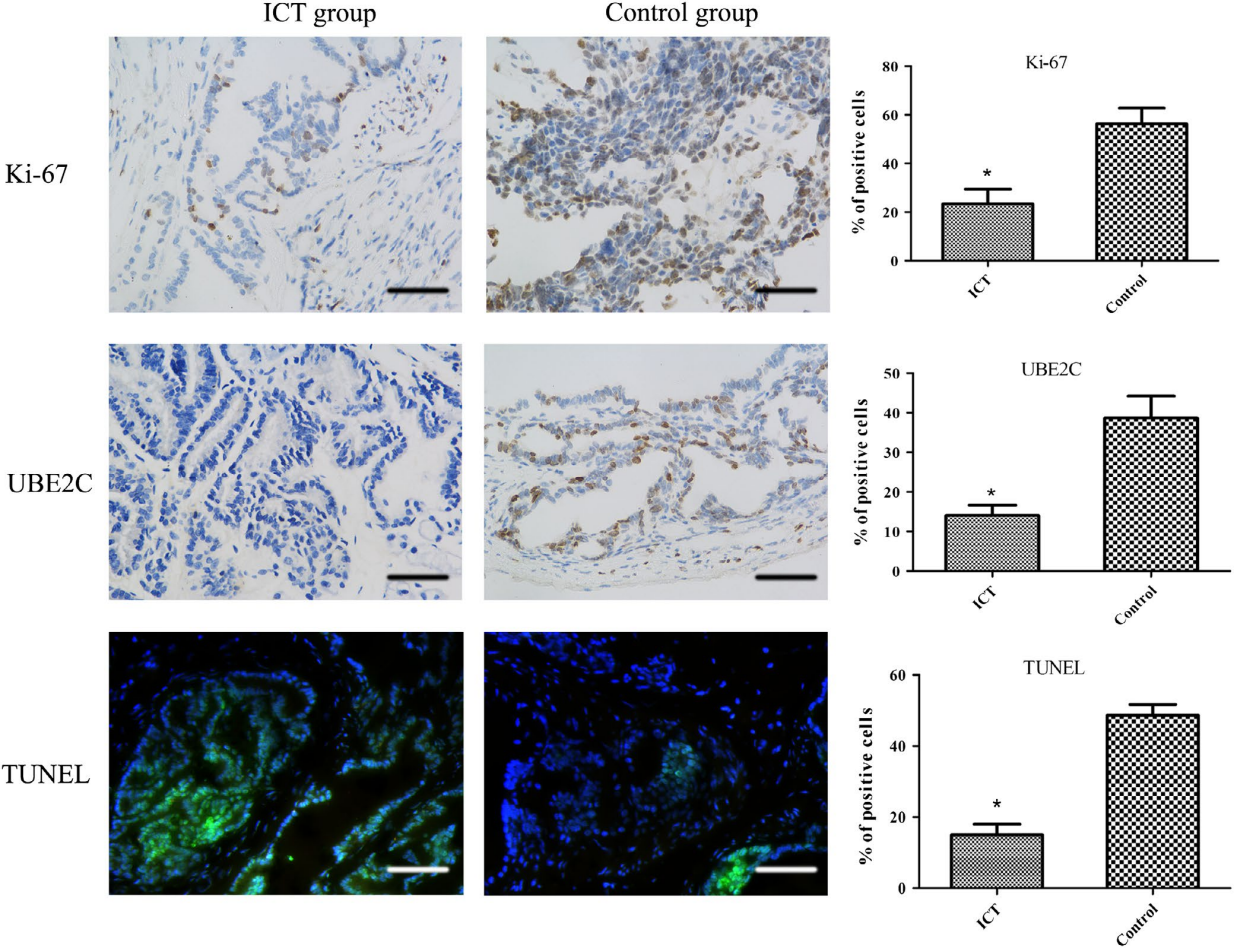
Representative images of tumor sections from TRAMP mice with Ki‐67, UBE2C or TUNEL staining (magnification, 400×; scale bar, 100 μm). *P < .05, relative to the respective control cells

Considering UBE2C expression was markedly upregulated in PCa cell lines and was associated with cell death and cell proliferation, we then assessed the UBE2C protein expression and cell proliferation marker Ki‐67 in PCa tissues of TRAMP mice from the ICT treatment group and the control group. Two mice from the ICT group and eight from the control group that died prematurely were excluded. Significantly lower expressions of UBE2C were identified in PCa tissues from the mice of ICT group compared with those of the control group. TUNEL assays also showed that ICT treatment enhanced the apoptotic cell death in PCa tissues in comparison with the untreated group (Figure 2). Furthermore, we investigated the effect of ICT treatment on cell proliferation in the PCa tissues. Our results showed markedly fewer proliferative (Ki67 positive) cells in the ICT treatment group compared with the control group (Figure 2). Taken together, our results indicate that ICT significantly reduced the UBE2C protein expression and inhibited PCa cell proliferation.

### 3.3 ICT suppresses PCa cell proliferation by G1 phase arrest

**Figure 3:**
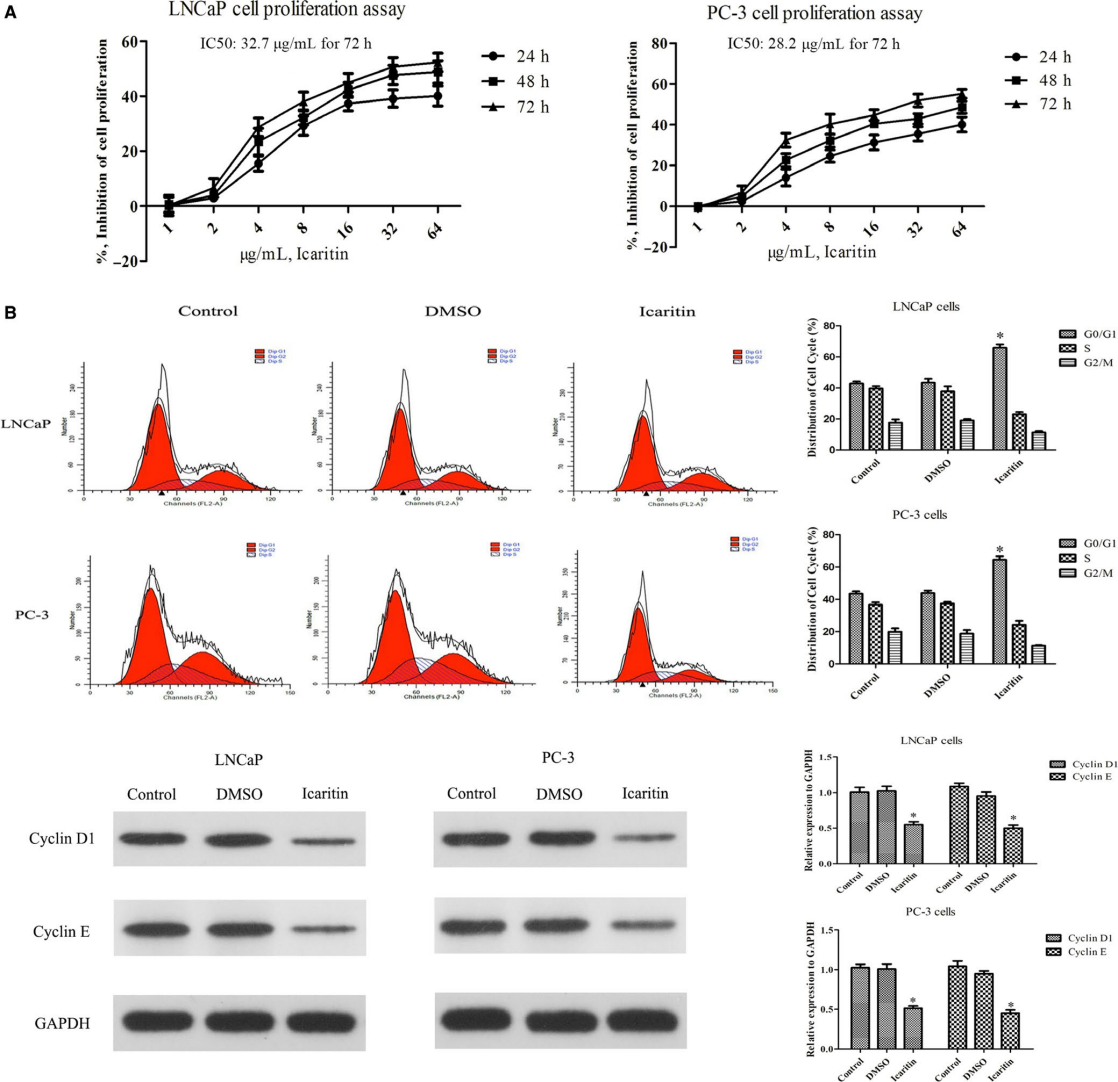
Icaritin (ICT) inhibits cell proliferation by inducing a strong G1 phase arrest in human LNCaP and PC‐3 cell lines. A, Human LNCaP and PC‐3 cells were treated with 0‐64μg/ml ICT for 24, 48 or 72 h, and cell‐inhibition rates were analyzed by the CCK‐8 assay. Results are expressed as a percentage of control levels. Data are represented by mean ± SD (three experiments). B, Cell cycle analysis of LNCaP cells treated with blank, DMSO or ICT for 72 h. PI fluorescence pattern was applied for cell cycle distribution. The bar graph shows the percentage of cell cycle distribution for each treatment group. The expression levels of two G1 phase‐related proteins (cyclin D1 and cyclin E) were evaluated by western blot assay. GAPDH was used as a loading control. *P < .05, relative to the respective control cells

To further investigate the mechanism of antitumor efficacy of ICT, human PCa cell lines (LNCaP and PC‐3) were used in following experiments. Both PCa cell lines were treated with ICT at different concentrations and cell growth was assessed using the CCK‐8 assay. Our results showed that ICT effectively inhibited cell proliferation in a dose‐dependent manner (Figure 3A). The best optimum dose of ICT was observed 32 μg/mL, which effectively inhibited LNCaP and PC‐3 cell proliferation with the inhibition rates of 49.73 ± 3.36% and 57.21 ± 4.05%, respectively. The IC50 of ICT in LNCaP and PC‐3 cells was determined as 32.7 and 28.2 μg/mL, respectively (Figure3A). Owing to the strong growth inhibition effect of ICT, we then analyzed its possible inhibitory effect on cell cycle progression. The cell cycle of LNCaP and PC‐3 cells was determined by flow cytometry under either DMSO vehicle control or the IC50 value of ICT for 72 hours. The results showed that ICT significantly induced G1 arrest in PCa cells after 72 hours of ICT treatment. Moreover, ICT evidently reduced the expression levels of G1 phase‐related proteins, such as cyclin D1 and cyclin E (Figure 3B). Thus, ICT could inhibit PCa cell proliferation by G1 phase arrest.

### 3.4 ICT induces cell apoptosis and inhibits the invasion ability of PCa cell lines

**Figure 4:**
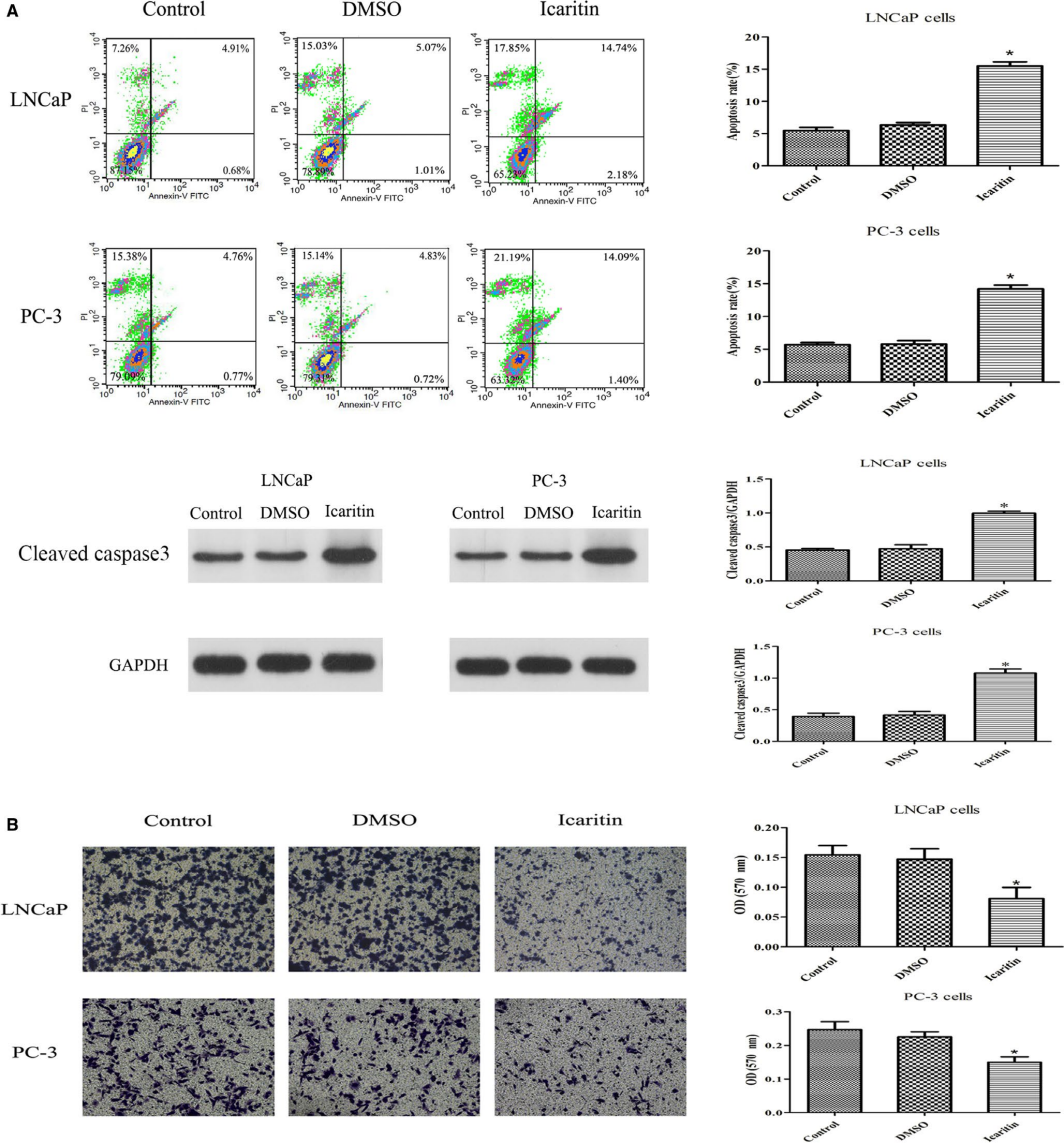
Effect of icaritin (ICT) treatment on cell apoptosis and invasion ability in PCa cell lines. A, Cell apoptosis rate of PCa cells were measured by flow cytometry. Cells were treated (72 h) at indicated doses, harvested, and stained with annexin‐V FITC and PI. Cells apoptosis rate was analyzed in each group. The protein level of cleaved caspase 3 was significantly increased by ICT. B, Representative images of PCa cells treated with each group by a transwell assay photographed from the light microscope (magnification, 100×). Transwell assay was performed to assess the cell invasion of each group. *P < .05, relative to the respective control cells

To test whether ICT had effects on PCa cell apoptosis and invasion, we then employed TUNEL and transwell assay in ICT‐treated PCa cells. After LNCaP or PC‐3 cells were treated at the IC50 of ICT (32.7 or 28.2 μg/mL, respectively) for 72 hours, a significantly higher apoptosis rate was detected in the ICT‐treated groups, along with the upregulated protein level of cleaved caspase 3, which is a marker protein of cell apoptosis (Figure 4A). In addition, transwell assays were performed to assess the cell‐invasion ability. PCa cells in complete medium were treated with either DMSO vehicle control or 35 μg/mL of ICT for 72 hours. We found that ICT significantly inhibited the invasion ability of LNCaP and PC‐3 cells compared with their control groups (Figure 4B).

### 3.5 Knockdown of UBE2C inhibited the proliferation and invasion of PCa cells

**Figure 5:**
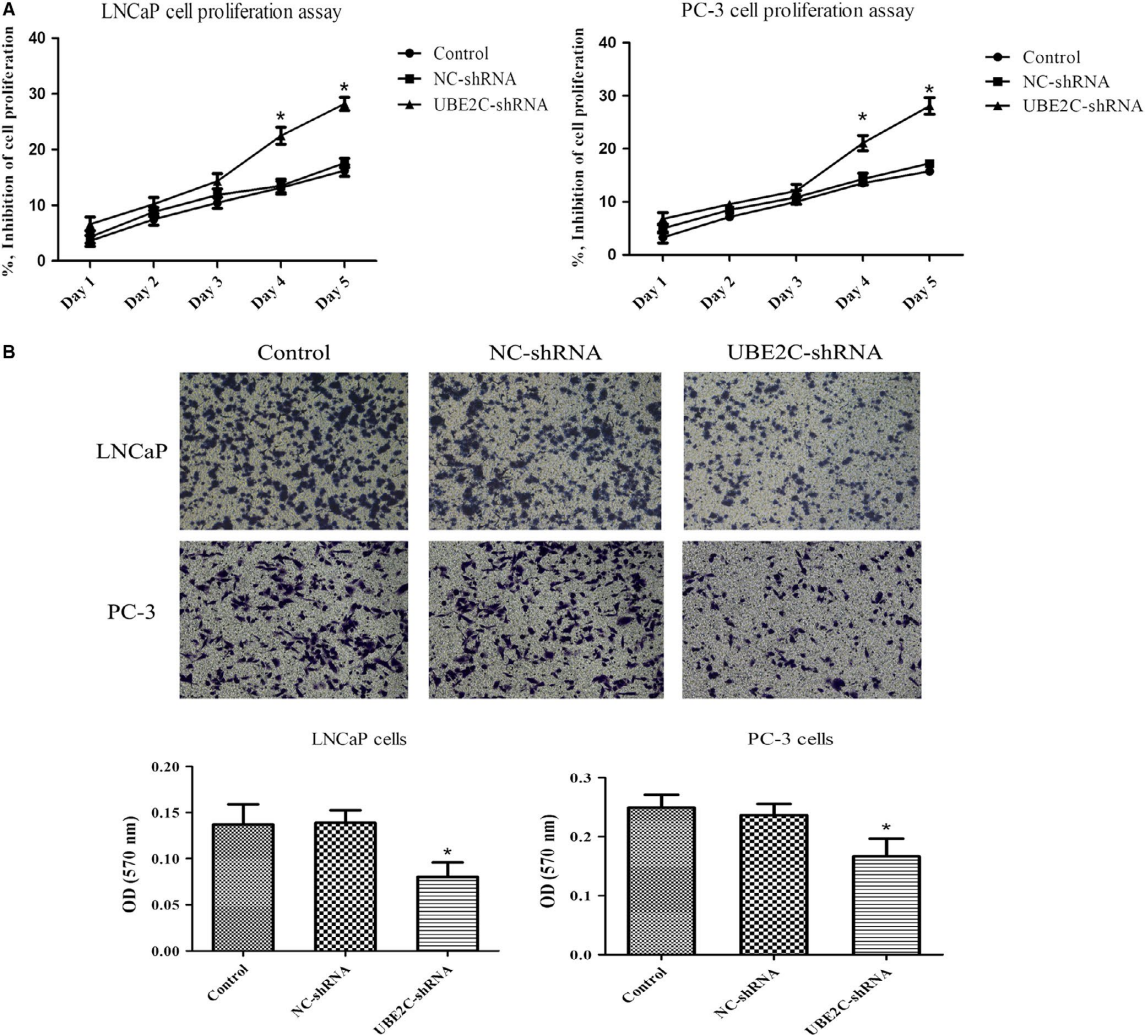
UBE2C is essential for the proliferation and invasion of PCa cells. A, Cell‐inhibition rates were measured by the CCK‐8 assay for 1‐5 days, after treatment with blank control, NC‐shRNA, or UBE2C‐shRNA. B, Representative images of PCa cells treated with blank control, NC‐shRNA, or UBE2C‐shRNA, by a transwell assay photographed from the light microscope (magnification, 100×). *P < .05, relative to the respective control cells

Lack of direct evidence showing UBE2C drives cell proliferation and invasion in PCa, we investigated the carcinogenic role of UBE2C in the human PCa cell lines LNCaP and PC‐3. The cell proliferation was evaluated using a CCK‐8 assay after knockdown of UBE2C. As expected, PCa cell proliferation rates were significantly slower than those of the control groups after UBE2C knockdown (Figure 5A). Meanwhile, a transwell assay was used to detect the ability of cell invasion, and we observed significantly fewer cells invaded into the lower surface of the membrane through Matrigel after UBE2C knockdown (Figure 5B). These results indicated that UBE2C was the key factor in proliferation and invasion of PCa cells.

### 3.6 MiR‐381‐3p directly suppresses UBE2C expression in PCa cells

**Figure 6:**
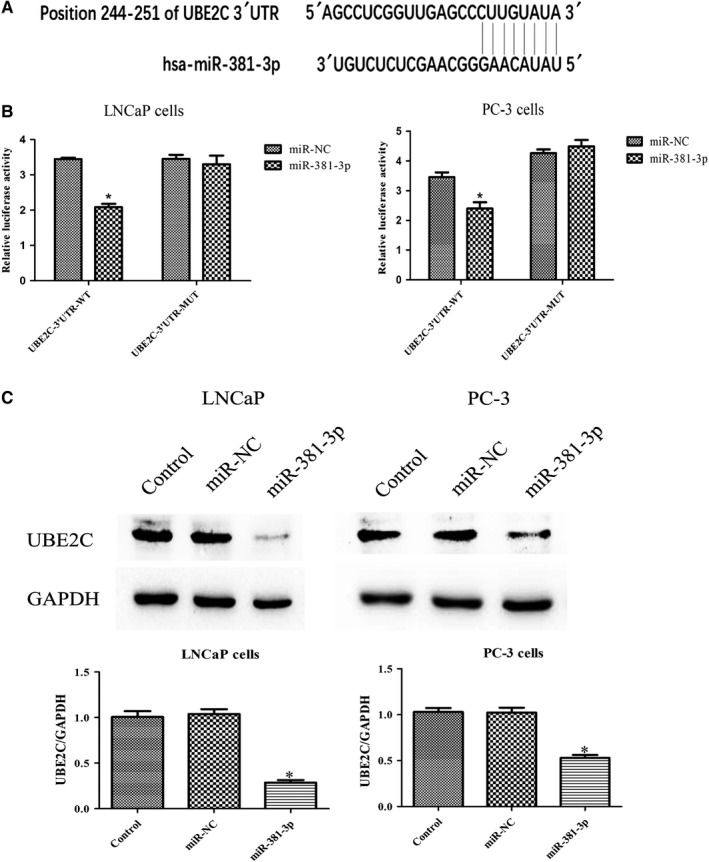
UBE2C is a direct target of miR‐381‐3p in PCa cells. A, The potential miR‐381‐3p binding sites of wild‐type UBE2C 3′UTR. B, The relative luciferase activity was detected in LNCaP and PC‐3 cells co‐transfected with miR‐381‐3p (or miR‐NC) and reporter plasmid carrying wild‐type or mutant UBE2C 3′UTR. C, Western blot assay was performed to determine the expression of UBE2C protein in LNCaP and PC‐3 cells either without transfection (blank control), or transfected with miR‐NC or miR‐381‐3p. *P < .05, relative to the respective control cells

Previous studies showed UBE2C is regulated by variety of miRNAs. To determine whether UBE2C was regulated by miRNAs in PCa, bioinformatic analyses were performed to identify the miRNA regulator of UBE2C gene. As indicated by the TargetScan, UBE2C mRNA has one theoretical miR‐381‐3p binding site within the 3′‐untranslated regions (3′UTR; Figure 6A). A luciferase reporter assay was conducted to confirm whether miR‐381‐3p is a regulator of UBE2C in PCa cells. As shown in Figure 6B, co‐transfection with miR‐381‐3p remarkably decreased the luciferase activity of the reporter plasmid carrying the wild‐type UBE2C 3′UTR. However, the suppressive effect was eliminated when the miR‐381‐3p binding sequence in the UBE2C 3′UTR was mutated. In addition, we verified these results by detecting UBE2C protein expression. After overexpression of miR‐381‐3p, endogenous UBE2C was significantly downregulated in both LNCaP and PC‐3 cells (Figure 6C). Taken together, miR‐381‐3p was a direct regulator of UBE2C in PCa cells. It can be deduced that miR‐381‐3p inhibited the proliferation of PCa cells by targeting UBE2C.

### 3.7 ICT downregulates UBE2C expression by upregulating miR‐381‐3p

**Figure 7:**
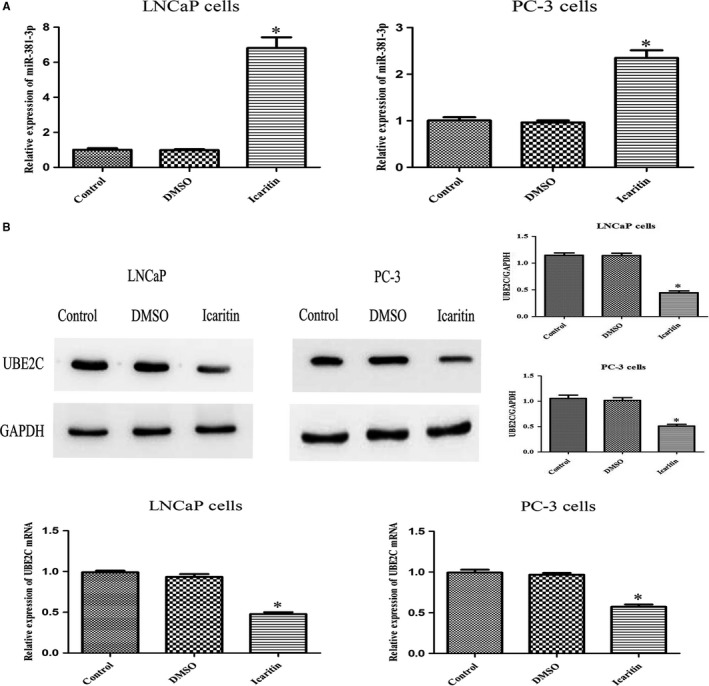
Effect of icaritin (ICT) treatment on miR‐381‐3p and its target gene UBE2C in PCa cell lines. A, LNCaP and PC‐3 cells were treated with blank, DMSO, or ICT. After 72 h, the expression of miR‐381‐3p was detected by qRT‐PCR; U6 snRNA served as an internal control. B, Western blot assay and real‐time PCR were performed to determine the expression of UBE2C protein and mRNA levels in LNCaP and PC‐3 cells treated with blank, DMSO, or ICT. *P < .05, relative to the respective control cells

After identifying UBE2C as a target gene of miR‐381‐3p, we next sought to determine whether ICT could regulate miR‐381‐3p and its target gene UBE2C. We measured the relative expression of miR‐381‐3p in PCa cells treated with either DMSO vehicle control or ICT. The results showed that ICT upregulated the expression of miR‐381‐3p in both LNCaP and PC‐3 cells as compared to the DMSO control group (Figure 7A). In addition, ICT significantly inhibited both protein and mRNA expression of UBE2C in PCa cells (Figure 7B). Collectively, ICT suppressed malignant biological behavior by regulating miR‐381‐3p and its target gene UBE2C in PCa cells.

## 4 DISCUSSIONS

It is evitable that majority of patients receiving androgen‐deprivation therapy will develop to androgen‐independent prostate cancers. In recent years, the serious adverse effects as well as acquired resistance to currently available chemotherapeutic agents became a universal challenge for oncologists. Alternatively, in the recent years various Chinese medicinal herbs have been widely applied in clinical treatment for cancer therapy. A previously published study illustrated that ICT could inhibit PC‐3 cell growth by inducing G1 phase arrest.^27^ Furthermore, ICT was also found to inhibit AR signaling in human PCa cells.^28^ Herein, we evaluated the antitumor action of ICT both in vivo in TRAMP mice and in vitro in human PCa cell lines, in order to fully assess the potential inhibitory mechanism of ICT on the occurrence and progression of PCa. In this study, the putative target and biological mechanism of ICT on PCa regression were also determined both in vivo and in vitro.

Previously, it has been reported that Prostatic intraepithelial neoplasia (PIN) and invasive prostate adenocarcinoma can be detected in TRAMP mice at 10‐12 and 18‐20 weeks of age, respectively. In addition, almost all the TRAMP mice develop PCa, and even some may have tumor metastasis by the age of 30‐36 weeks.^29^ In this study, we investigated the direct impact of ICT on the growth inhibition of tumor cells using Ki‐67 staining. Ki‐67 is a nuclear protein that is associated with cellular proliferation and is also an independent predictor of metastasis and a cause‐specific mortality of PCa.^30^, ^31^, ^32^ Our preliminary results demonstrated that ICT significantly inhibited the expression level of Ki‐67 in PCa tissue, suggesting the growth inhibitory function of ICT on PCa cells. Meanwhile, we also analyzed the expression of UBE2C in PCa tumor tissues. UBE2C is essential for mitotic cyclins and regulating anaphase‐promoting complexes.^33^, ^34^ Overexpression of UBE2C may cause chromosome missegregation and change the cell cycle profile, which facilitates cell proliferation.^6^, ^35^ Thus, UBE2C would be a potential biomarker for tumor diagnosis or prognostic determination. Herein, our results demonstrated that ICT treatment inhibit tumor development and progression of PCa in TRAMP mice by downregulating UBE2C expression in prostatic cancer tissues.

In addition, ICT inhibits PCa growth by downregulating cell proliferation, cell cycle, and cell invasion under the influence of UBE2C function. Furthermore, the retroviral vector‐derived UBE2C shRNA was applied to knock down the UBE2C expression, which confirmed the critical role of UBE2C in cell proliferation and invasion. The downregulation of UBE2C expression upon ICT treatment also confirmed the previous finding that UBE2C is important in the tumor cell growth in PCa cells.

MicroRNAs play critical roles in the cancer pathogenesis through regulation of various biological processes. UBE2C was identified as a direct target gene of miR‐381‐3p in PCa cells. We found that treatment of ICT downregulate tumor promoting oncogene UBE2C by upregulating its regulatory microRNA, mir‐381‐3p, in PCa cells. Our results provide strong evidence that ICT could be applied as a novel therapeutic agent in PCa treatment through the putative molecular targeting.

In 2007, Porkka et al reported the specific expression of miRNA in patients with PCa for the first time.^36^ Currently, various miRNAs from circulating blood can be used as markers for the diagnosis and prognosis of PCa patients.^37^, ^38^, ^39^ Herein, the luciferase assay indicated that miR‐381‐3p targeted UBE2C directly in both LNCaP and PC‐3 cells. This finding was further confirmed by immunoblot assays. Although one miRNA can regulate multiple target genes, the inhibitory functions of miR‐381‐3p in PCa should be attributed, at least in part, to the suppression of UBE2C. For instance, miR‐21 can induce chemoresistance to docetaxel in PC‐3 cells.^40^ Another study found that downregulation of miR‐205 and miR‐31 could confer resistance to chemotherapy‐induced apoptosis in PCa cells.^41^ In line of these findings, our results showed that ICT can inhibit the malignant transformation of PCa by upregulating miR‐381‐3p expression levels, which suppressed the expression and function of oncogenic UBE2C. Based on these results, further study could be performed in a combinatorial approach using ICT along with conventional chemotherapeutic agents to treat TRAMP mice and PCa cells in order to determine the synergistic efficacy of ICT with miR‐381‐3p in prostate cancer therapy.

In summary, our studies depicted the novel insight into the biological function of ICT in the suppression of malignant transformation of PCa by altering the expression and function of a chemoresistance‐promoting oncogene UBE2C through miR‐381‐3p regulation. Based on our experimental results, we envision that ICT might serve as a novel therapeutic alternative for CRPC.
